# Vegetation fire smoke, indigenous status and cardio-respiratory hospital admissions in Darwin, Australia, 1996–2005: a time-series study

**DOI:** 10.1186/1476-069X-7-42

**Published:** 2008-08-05

**Authors:** Ivan C Hanigan, Fay H Johnston, Geoffrey G Morgan

**Affiliations:** 1 School for Environmental Research, Charles Darwin University, Ellengowan Drive, Darwin, 0909, Northern Territory, Australia; 2 Menzies School of Health Research, Charles Darwin University, Ellengowan Drive, Darwin, 0909, Northern Territory, Australia; 3 Menzies Research Institute, University of Tasmania, Private Bag 23 Hobart 7001, Tasmania, Australia; 4 North Coast Area Health Service and Department of Rural Health (Northern Rivers) University of Sydney, 55–61 Uralba Street, Lismore, 2480, New South Wales, Australia

## Abstract

**Background:**

Air pollution in Darwin, Northern Australia, is dominated by smoke from seasonal fires in the surrounding savanna that burn during the dry season from April to November. Our aim was to study the association between particulate matter less than or equal to 10 microns diameter (PM_10_) and daily emergency hospital admissions for cardio-respiratory diseases for each fire season from 1996 to 2005. We also investigated whether the relationship differed in indigenous Australians; a disadvantaged population sub-group.

**Methods:**

Daily PM_10 _exposure levels were estimated for the population of the city from visibility data using a previously validated model. We used over-dispersed Poisson generalized linear models with parametric smoothing functions for time and meteorology to examine the association between admissions and PM_10 _up to three days prior. An interaction between indigenous status and PM_10 _was included to examine differences in the impact on indigenous people.

**Results:**

We found both positive and negative associations and our estimates had wide confidence intervals. There were generally positive associations between respiratory disease and PM_10 _but not with cardiovascular disease. An increase of 10 μg/m^3 ^in same-day estimated ambient PM_10 _was associated with a 4.81% (95%CI: -1.04%, 11.01%) increase in total respiratory admissions. When the interaction between indigenous status and PM_10 _was assessed a statistically different association was found between PM_10 _and admissions three days later for respiratory infections of indigenous people (15.02%; 95%CI: 3.73%, 27.54%) than for non-indigenous people (0.67%; 95%CI: -7.55%, 9.61%). There were generally negative estimates for cardiovascular conditions. For non-indigenous admissions the estimated association with total cardiovascular admissions for same day ambient PM_10 _and admissions was -3.43% (95%CI: -9.00%, 2.49%) and the estimate for indigenous admissions was -3.78% (95%CI: -13.4%, 6.91%), although ambient PM_10 _did have positive (non-significant) associations with cardiovascular admissions of indigenous people two and three days later.

**Conclusion:**

We observed positive associations between vegetation fire smoke and daily hospital admissions for respiratory diseases that were stronger in indigenous people. While this study was limited by the use of estimated rather than measured exposure data, the results are consistent with the currently small evidence base concerning this source of air pollution.

## Background

Associations between daily hospital admissions for cardio-respiratory diseases and particulate matter less than or equal to 10 microns in aerodynamic diameter (PM_10_) have been described in many settings worldwide including North America, Europe, Asia and Australia [1]. In large cities, where the vast majority of research has been conducted, fossil fuel combustion in industry and transport are major sources of PM_10_. However, depending on the setting, there are potential contributions from a range of other sources including crustal particles and biomass combustion such as forest fires and wood fuels [2]. The relative effects of different sources of particulate pollution on adverse heath outcomes, and differences in these effects across population sub-groups, remain major gaps in the currently available evidence [1]. In particular, the relative role of particulates derived from biomass as opposed to fossil fuel combustion remains unclear, although two empirical studies of PM_10 _derived from vegetation fires [3,4], and one review of studies examining PM_10 _from wood smoke [5] all observed that the magnitude of associations with respiratory outcomes is greater when PM was derived from biomass combustion. However, studies examining a single source of ambient PM_10 _are infrequent because of the difficulty finding a site without a mixture of various pollutants, and the complexity of apportioning contributions from different sources [2,6]. A few epidemiological studies have apportioned total PM according to a range of sources (such as biomass, crustal and motor vehicles) and have found a range of different clinical outcomes were associated with different exposure sources [7,8].

The city of Darwin enables the health effects of vegetation fire smoke to be assessed because the source of the PM pollution is due almost entirely to fire smoke. Particulate matter derived from biomass combustion has been identified as an increasing and unregulated source of outdoor air pollution. The use of wood burning for domestic heating is increasing in several countries [9,10], while the frequency and severity of uncontrolled vegetation fires is increasing the world over [11]. Vegetation fires generate pollution episodes across wide geographic areas, and major population centers are frequently affected [12].

In Australia, the increasing use of deliberate fuel reduction burns as hazard reduction activities to avert major fire disasters is becoming more controversial in the light of the evidence of adverse health impacts of particulate air pollution [13].

The tropical city of Darwin (Latitude: -12.462, Longitude: 130.842) provides an opportunity to specifically examine the health associations of vegetation fire smoke. Here 50–70% of the surrounding savanna burns annually during the 8 month dry season between April and November [14]. The months from December until March are referred to as the wet season when approximately 80% of Darwin's average annual total rain falls. Due to the rain, fires only occur during the dry season and the smoke from these fires are the source of 95% of measured PM_10 _in the city [15]. There are no other important sources of air pollution such as traffic or industry and so PM is negligible during the wet season [16]. A comprehensive air quality study was conducted in the year 2000 [15] and the average concentrations of other pollutants including ozone, sulfur dioxide and nitrogen dioxide are negligible.

During the dry season, prevailing south-easterly winds bring vegetation fire smoke over Darwin from a large region of savanna. The lower atmosphere of the airshed is characteristically stable during dry seasons and there is a persistent inversion at about 3000 meters [17]. These conditions produce similar concentrations of ambient PM_10 _across the city. This was validated in 2005 when PM_10 _measurements at two monitors located 25 km to the west and south were shown to be of similar magnitude and highly correlated with the primary monitor [17]. This evidence shows the monitor values for PM_10 _are representative of the community's exposure.

The pattern of vegetation burning remained consistent throughout the study period. This was demonstrated by analysis of satellite data which have confirmed the ongoing regional and seasonal nature of annual landscape fires [18] and by an air quality monitoring campaign conducted 25 km north-west of Darwin in the mid 1990s [16].

Darwin has a population of approximately 110,000 people and also provides an opportunity to examine the relative impact on indigenous Australians, a high-risk population subgroup comprising 11% of the population of Darwin [19]. Socio-economic disadvantage, chronic cardio-respiratory diseases and diabetes have all been shown to modify the effect of particulate air pollution on health outcomes [20]. Indigenous Australians have a high prevalence of all these health risks and have been recognized as being likely to be at much greater risk from poor air quality than other Australians [21]. This has been stated as a priority for Australian public health research [22].

A previous case-crossover study of the hospital admissions and observed PM_10 _in Darwin showed a positive association with respiratory diseases, and disproportionately higher effect estimates in indigenous people [23]. That study had limited statistical power as Darwin's population is relatively small and only three years of air quality data were available for analysis. Here we attempt to address these limitations by using PM_10 _estimations over a 10-season period using a previously validated predictive model based on visibility records [17] and using the alternative method of time series modeling [24].

## Methods

### Study period

We examined data for the fire seasons between the 1^st ^of April and 30^th ^of November each year from 1996 to 2005. This period corresponds with the tropical dry seasons, which is characterised by constant savanna fires and regional smoke of fluctuating intensity as described above. Wet seasons were excluded because 80% of Darwin's average annual rainfall (1700 mm) falls during this period, landscape fires are absent and airborne PM is consequently negligible [15,16].

### Outcome measures

De-identified individual records of all persons admitted to the Royal Darwin Hospital for respiratory or cardiovascular conditions were provided by the Northern Territory Department of Health and Community Services. Elective admissions were excluded from the analysis. This is the only hospital in Darwin and services the entire population of the city and surrounding areas.

Principal diagnosis, indigenous status and primary residence were recorded on discharge from the hospital. Patients whose primary residence was not in Darwin were excluded.

Data were extracted by their assigned principal diagnosis codes classified according to the International Classification of Diseases (ICD) codes. In 1999 there was a change in the coding system used to assign diagnoses from the ICD edition 9 to edition 10. A concordance list produced by the New Zealand Health Information Service was used to marry the diagnosis codes across these two classification systems.

Time series of daily admissions were constructed for each 8-month fire season between 1996 and 2005 for the following diagnosis groups: Total Cardiovascular (ICD9 = 390–459, ICD10 = I00-I99), Ischemic Heart Disease – IHD (ICD9 = 410–414, ICD10 = I20-I25), Total Respiratory (ICD9 = 460–519, ICD10 = J00-J99), Asthma (ICD9 = 493, ICD10 = J45-J46), Chronic Obstructive Pulmonary Disease – COPD (ICD9 = 490–492, 494–496, ICD10 = J40-J44, J47, J67) and Respiratory Infections (ICD9 = 461–466, 480–487, 514, ICD10 = J00-J22).

Ethical approval was gained from the Human Research Ethics Committees of the Northern Territory Government Department of Health and Community Services, the Menzies School of Health Research and the Charles Darwin University.

### Exposure measures

We used a predictive model for deriving exposure measures for ambient PM_10 _from visibility data because of the limited availability of empirical air quality data. Vegetation fire smoke is the main determinant of visibility during the dry seasons as rain and fog are rare events and there are no other important sources of air pollution. PM_10 _was measured in Darwin during the years 2000, 2004 and 2005 and at Charles Point, 25 km west of central Darwin, during 1995. Data for the years 2000 and 2004 were used to develop the model, while data from 2005 and 1995 were used to assess the performance of the model. In addition, predicted peaks in PM_10 _during 2000 and 2001 were mapped against bushfire activity records for this period. The development and validation of this model were described in detail by Bowman *et al *[17] and below we summarise how this was done. Insufficient measurements were available for PM_2.5 _to develop a predictive model for this size class of PM.

### Data used for the development of the model

In 2000 the PM_10 _was measured using a Tapered Element Oscillating Microbalance (Rupprecht and Patashnick series 1400a, East Greenbush, NY, USA), which provided continuous PM_10 _loadings (μg/m^3^) with a 30-minute time resolution. This was centrally located at the Commonwealth Scientific and Industrial Research Organization research site at Darwin airport. Observations for 2004 and 2005 were obtained using a sequential air sampler (Rupprecht and Patashnick Partisol plus, model 2025, East Greenbush, NY, USA), which provided 24-hour gravimetric measures of PM_10_(μg/m^3^). This monitor was located at the Charles Darwin University, 7 km from the Darwin airport. Previous studies have demonstrated a high correlation and similar magnitude of daily PM_10 _measured at these sites within Darwin [15].

In 1995 a monitor was located at Charles Point, 25 km west of central Darwin. PM_10 _was measured gravimetrically using stacked filter units with an inlet that sampled particles less than or equal to 10 μm in diameter. These units sampled continuously for a period of 3 to 5 days, giving a 3- to 5-day average particulate concentration for each day within that sampling time [16]. During 2005, Bowman *et al *[17] assessed how PM measured at this site, compared with PM measured in the city by placing parallel monitors at the two locations. Daily PM_10 _over a two month period were highly correlated (r^2 ^= 0.75), although the overall mean PM_10 _was lower at Charles Point than in Darwin (20.89 vs 23.85 μg/m^3^). We judged this correlation to be sufficiently high to justify the use of the 1995 data from Charles Point as a secondary independent validation of the predicted PM for Darwin.

Daily visibility and meteorological data were collected by the Australian Bureau of Meteorology at the Darwin Airport, located in the centre of the city. These data included precipitation in mm in the preceding 24 h before 0900 hours (local time); average total cloud amount in eighths; maximum air temperature degrees Celsius; averaged relative humidity percentage (from observation made at 0900, 1200 and 1500 hours); and average wind speed in km/h (from observations made at 0900, 1200 and 1500 hours). Average visibility in meters was derived from observations made at 0000, 0300, 0600, 0900, 1200, 1500, 1800 and 2100 hours. Visibility measurements have been made at this location since the 1950s, following the international standard practice of determining whether or not reference objects at known distances from the site were visible to the human observer.

### Development of the model

The model was constructed using a training dataset of visibility, meteorological observations and daily PM_10 _for the dry season months of 2000 and 2004. Predictive models of daily PM_10 _were developed using Gaussian linear mixed modelling. To overcome the possibility of systematic changes associated with the progression of the dry season, 'month' was included as a random effect. A range of candidate models were assessed and final model was selected using the Akaike Information Criterion (AIC). The final model was as follows:

PM_10 _= (73.86 - monthly correction) + (-1.511 × visibility) + (-0.113 × rainfall) + (-0.262 × relative humidity)

### Assessment of the model

**Figure 1:**
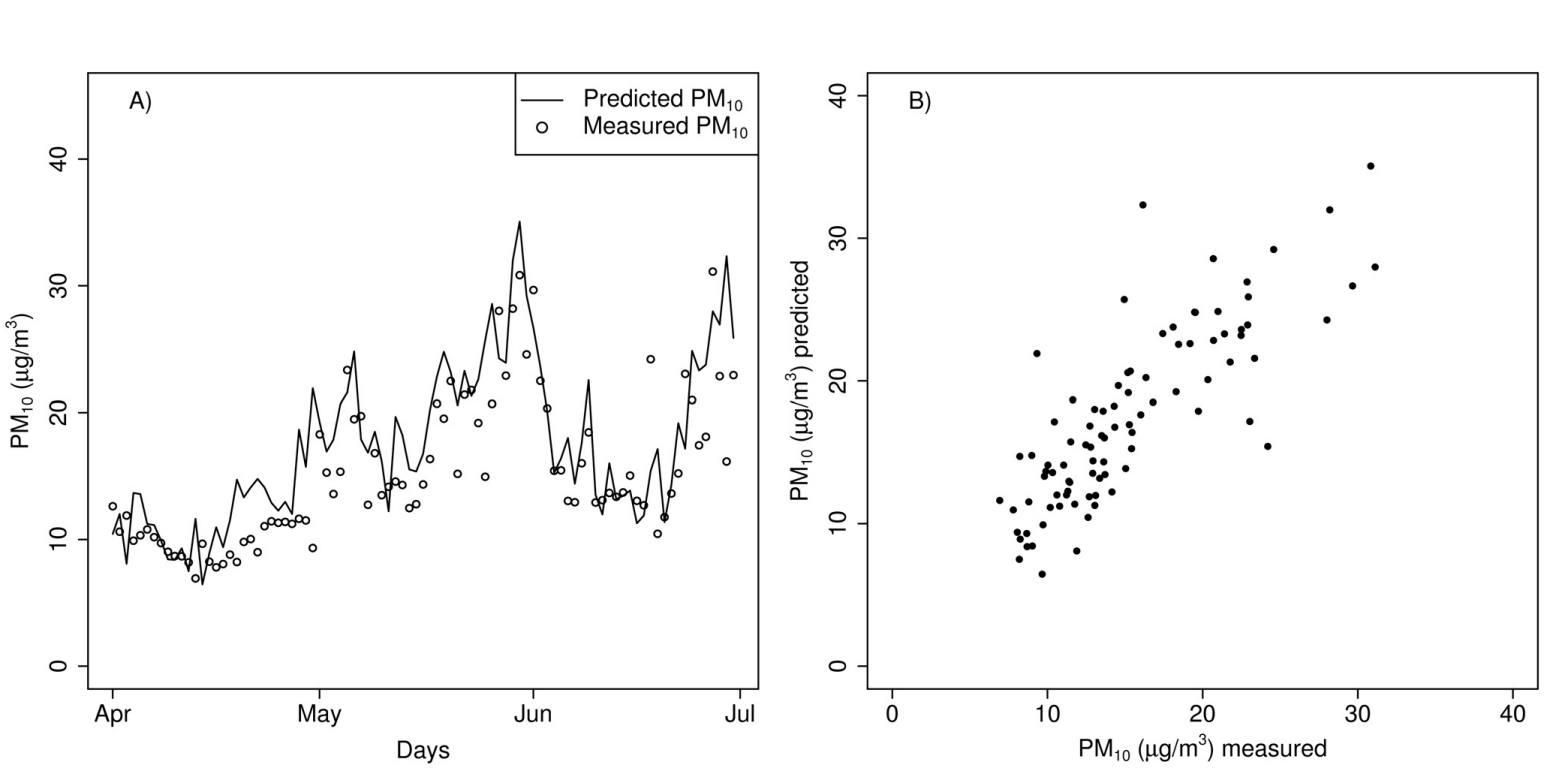
**Model predictions of ambient PM_10 _against the measured data in 2005**. Comparison of model daily predictions of ambient PM_10 _(μg/m^3^) using visibility with measured data withheld from modeling for use as validation dataset: A) superimposed to show day-to-day variation and B) as a scatter plot to show correlation (r^2 ^of 0.68, slope = 0.90). Observed PM_10 _is included for comparison purposes only, the study used predicted PM_10 _values only.

**Figure 2:**
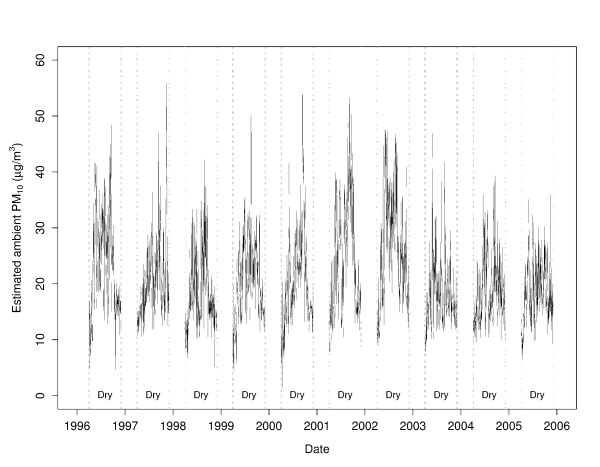
**Daily estimated ambient PM_10 _for Darwin during each 8-month dry season, 1996–2005**. Ambient PM_10 _(μg/m^3^) was estimated from visibility and weather data. No estimates were made for the 4-month wet seasons.

Daily predicted estimates were validated against observations from a 91 day period (April – June) in 2005 which had been withheld from the training dataset used in model development. The measured observations for that period are shown superimposed on the predicted values in Figure 1, section A. The relationship between the predicted and measured ambient PM_10 _from 2005 is shown in Figure 2 section B. The predicted ambient PM_10 _correlated well with the observations with an r^2 ^of 0.68 and a slope of 0.90. The mean deviation between the predicted and measured values was -2 μg/m^3 ^with a standard deviation of 3.6. No adjustment was made for this small bias. Summary statistics for the estimated and true values in the validation dataset are shown in Table 1.

**Table 1:** Summary statistics for measured and predicted PM10 (μg/m3) from April – June 2005.

	Mean	Median	Minimum	Maximum
Measured	15.31	13.67	6.93	31.12
Predicted	17.42	16.40	6.45	35.07

The model was further tested in two ways. It was used to generate monthly averaged predictive values to compare with PM_10 _collected at Charles Point (a location to the north-west) during the 1995 dry season. These data were highly correlated (r^2 ^= 0.89). As expected from previous comparisons, the mean PM_10 _at Charles Point were slightly lower than that predicted for Darwin (17.6 vs 18.7 μg/m^3^). Finally, peaks in the predicted PM_10 _were mapped against both satellite records and written documentation of the date and location of significant fire activity held by the Bureau of Meteorology for the dry seasons of 2000 and 2001. Predicted peaks in PM_10 _were found to correlate well with both these records [17].

The estimated ambient PM_10 _levels from this predictive model for our study period are shown in Figure 2.

### Measurement of covariates for hospital data analysis

Mean daily relative humidity (percentage) and temperature (Celsius) measured at Darwin airport were provided by the Bureau of Meteorology. Three days of missing temperatures were imputed with the prior and subsequent days. Weekly influenza data (as a rate per 1000 consultations) were provided by the tropical influenza surveillance system network of sentinel General Practitioners (Northern Territory Department of Health and Community Services, Darwin). An epidemic was defined as periods during which influenza rates were greater than the 90^th ^percentile.

### Statistical modeling

Statistical approaches for analyzing time series data in air pollution studies continue to be refined [25-28]. Here we have followed the methods of the American Medicare Air Pollution Study [28] and the National Morbidity, Mortality and Air Pollution Study [29] by using over-dispersed Poisson generalized linear models with natural cubic splines for smoothed functions of time and meteorological variables. These authors suggested other studies reproduce their analyses using the same methods to increase the comparability of results of air pollution studies and have made their computer code available on the web for adaptation. We adapted Peng's code [30] to suit our data as follows: we included variables for relevant local factors including indigenous status, influenza epidemics, holidays, and the change between ICD editions but did not stratify by age because of the extremely low numbers of daily admissions this would create for some diagnosis groups.

Our regression models separately analyzed the association of same day estimated ambient PM_10 _and lags up to three days with daily admission counts for each diagnostic group. Potential confounding or modifying explanatory variables were included in all analyses using previously established protocols for air pollution health studies [31]. We included additional parameters to control for time varying factors including influenza epidemics and school holidays. Annual estimates of the populations of indigenous and non-indigenous Darwin residents were included as an offset in the model as the total population of Darwin grew by 10% during the study period.

We used an over-dispersed Poisson model of daily hospital admissions as follows:

log [E(Y_t_)] = β_1 _Lagged PM_10 _+ β_2 _Indigenous + ns(Time) + ns(AvDailyTemp) + ns(AvDailyTemp_Lag1-3_) + ns(RHumAv) + ns(RHumAv_Lag1-3_) + DOW + FluEpidemic + ICD10change + Holidays + offset(log(Population))

Where E(Y_t_) is the expected admission count on day t and 'ns' represents natural cubic splines. These variables, and the degrees of freedom (df) used in splines that represent them, are explained in Table 2.

**Table 2:** explanatory variables used in all models.

Variable	Description
Lagged PM_10_	Estimated ambient PM_10 _for each single-day lag 0, 1, 2 or 3 in (μg/m^3^)
Indigenous	An index of counts for indigenous status where indigenous = 1 and non-indigenous = 0
Time	Time in days, represented by a natural cubic spline with 40 df (4 df per dry season)
AvDailyTemp	Average daily temperature (calculated by averaging the max and min temperatures), in Degrees Celsius (°C), with 6df
AvDailyTemp_Lag1-3_	Moving three-day averages of daily temperatures (lags 1, 2 and 3), with 6df
RHumAv	Average daily relative humidity in percent (%) with 3df
RHumAv_Lag1-3_	Moving three-day averages of daily relative humidity (lags 1, 2 and 3), with 3 df
DOW	Day of the week. Factor with 7 levels
FluEpidemic	Influenza epidemics. Dummy for days above the 90th centile
ICD10change	The change between ICD editions. Dummy variable indicating the changeover
Holidays	Dummy variable for public holidays
Population	The estimated yearly population for indigenous or non-indigenous residents included as an offset

Because school holidays are likely to be related to rates of hospital admissions in children [31] these were included as a dummy variable for total respiratory admissions, asthma and respiratory infections as these conditions had a high proportion of children aged less than 15 years.

Finally, an interaction term between indigenous status and estimated ambient PM_10 _was added to the model to investigate the difference in the magnitude of the association in the two population sub-groups.

All analyses were conducted using the statistical software package R version 2.3.1 [32].

## Results

There were 2,410 days in the 10 dry seasons of our study period. There were 8,279 admissions during this period. The total numbers of hospital admissions (and proportion of patients under 15 years old) are given in Table 3, stratified by clinical grouping and indigenous status. Despite indigenous people representing 11% of the population of Darwin, they comprised 23% of these admissions.

**Table 3:** Emergency hospitalizations to the Royal Darwin Hospital for the dry seasons 1996–2005.

			Total population		Non-Indigenous admissions		Indigenous admissions		Percent < 15 yrs (total population)
Population in each group			109,478		97,887		11,591		

Diagnosis	ICD9	ICD10	Counts	Percentage	Counts	Percentage	Counts	Percentage	

Cardiovascular									
Total	390–459	I00-I99	3443	100%	2854	100%	589	100%	1%
IHD	410–414	I20-I25	1533	45%	1287	45%	246	42%	0%
Other	-	-	1910	55%	1567	55%	343	58%	2%
Respiratory									
Total	460–519	J00-J99	4836	100%	3551	100%	1285	100%	40%
Asthma	493	J45-J46	1008	21%	776	22%	232	18%	58%
COPD	490–492, 494–496	J40-J44, J47, J67	995	21%	753	21%	242	19%	1%
Infections	461–466, 480–487, 514	J00-J22	2409	50%	1681	47%	728	57%	53%
Other	-	-	424	9%	341	10%	83	6%	16%

Descriptive statistics for daily admissions in each disease category, estimated daily ambient PM_10 _and meteorological parameters are summarized in Table 4.

**Table 4:** Statistics for hospitalizations, estimated PM_10 _and weather in Darwin for dry seasons 1996–2005.

Diagnosis		Mean	Standard Deviation	Range
Cardiovascular	Total	1.4	1.2	6.0
	Indigenous	0.2	0.5	4.0
	Non-Indigenous	1.2	1.1	6.0
IHD	Total	0.6	0.8	5.0
	Indigenous	0.1	0.3	2.0
	Non-Indigenous	0.5	0.7	5.0
Respiratory	Total	2.0	1.5	10.0
	Indigenous	0.5	0.7	4.0
	Non-Indigenous	1.5	1.2	7.0
Asthma	Total	0.4	0.7	5.0
	Indigenous	0.1	0.3	2.0
	Non-Indigenous	0.3	0.6	3.0
COPD	Total	0.4	0.7	4.0
	Indigenous	0.1	0.3	2.0
	Non-Indigenous	0.3	0.6	4.0
Respiratory infections	Total	1.0	1.1	7.0
	Indigenous	0.3	0.5	3.0
	Non-Indigenous	0.7	0.9	5.0
Daily Estimated Ambient PM_10 _(μg/m3)		21.2	8.2	55.2
Daily Average Temperature (°C)		27.4	2.2	13.1
Daily Average Relative Humidity (%)		65.0	11.1	70.4
Influenza rates (weekly cases per 1000 consults for each day of the week)		13.2	12.3	82.4

Our modeling procedure used a sensitivity analysis similar to the method described by Dominici and colleagues [26] to select the optimal degrees of freedom for the smoothed function of time; to minimize bias in the estimates of the pollution coefficients. This sensitivity analysis was applied to the model for the estimated ambient PM_10 _lag with the greatest absolute t-value. We adjusted the degree of smoothing on the time variable by applying different values of a multiplier (α) that ranged from 0.2 to 3 times the degrees of freedom which had been chosen a priori [28,30]. The influence that this had on the effect estimate was assessed using the change in the mean squared error. Theoretically there is lower bias in the estimate caused by smoothing at higher values of α, but there is larger statistical uncertainty. We conservatively selected the optimal smoothing function for minimizing bias in the point estimate.

The point estimates and 95% Confidence Intervals (CI) for the association between hospital admissions with estimated ambient PM_10 _are reported here as the percentage change in the relative risk per 10 μg/m^3 ^change in exposure.

Initial modeling without the interaction between indigenous status and PM_10 _found a positive association for total respiratory admissions with same day estimated ambient PM_10 _(4.81%; 95%CI: -1.04%, 11.01%). The subgroups of respiratory infections, asthma and COPD all had positive associations with same day estimated ambient PM_10_. The small associations for all cardiovascular diseases and IHD were all negative or zero and not statistically significant. Due to small numbers in these groups the confidence intervals are wide.

**Figure 3:**
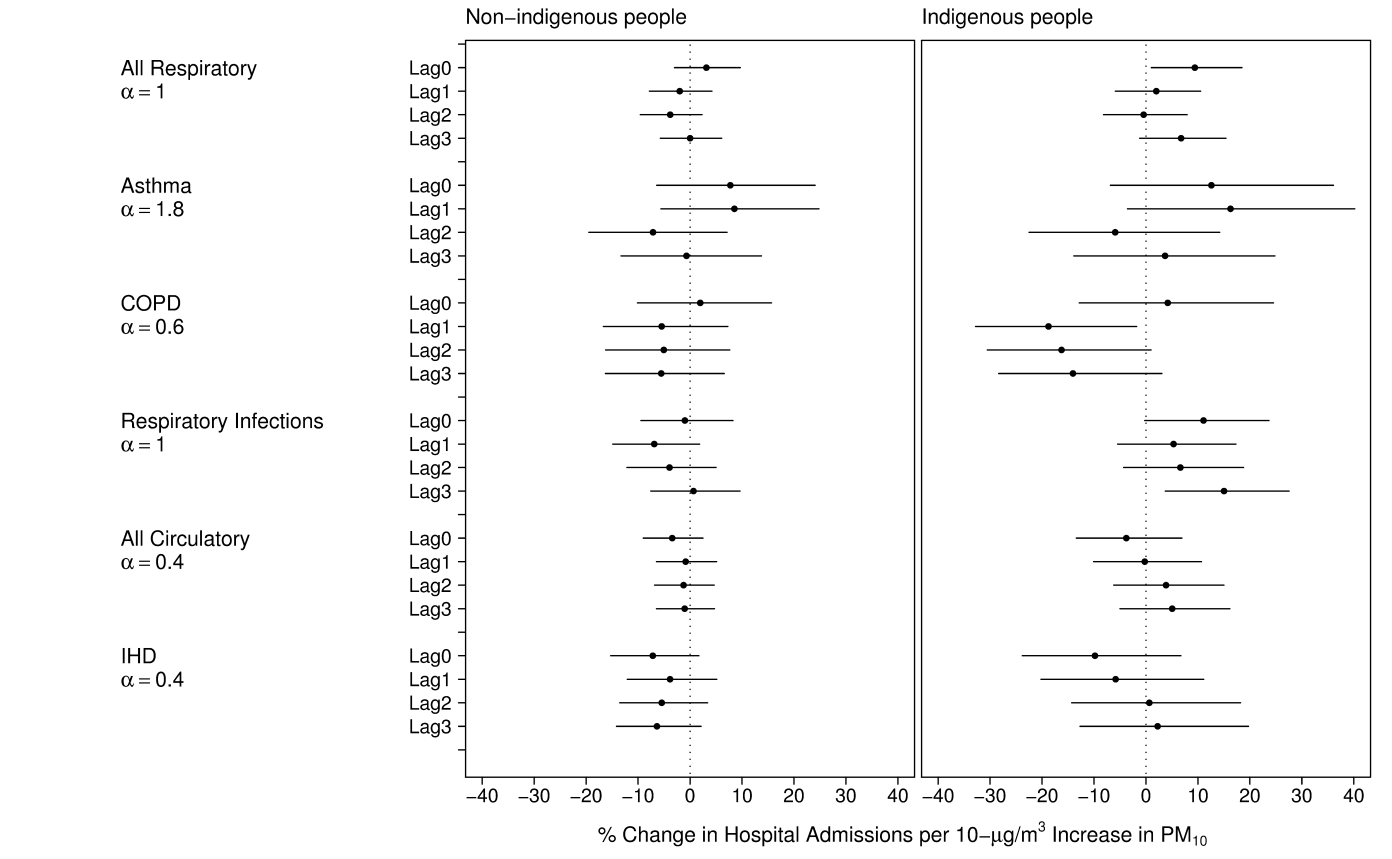
**Associations between hospitalizations for non-indigenous and indigenous people with estimated ambient PM_10_**. Point estimates and 95% confidence intervals for the association between hospital admissions for non-indigenous and indigenous people with estimated ambient PM_10 _in Darwin 1996–2005, as the percentage change in relative risk per 10 μg/m^3 ^rise in PM_10_. α represents the optimal level of a multiplication factor for the smooth function of time, selected using sensitivity analysis.

We then compared the effects for indigenous and non-indigenous people. Figure 3 shows the point estimates and 95% confidence intervals for the association between hospital admissions with estimated ambient PM_10 _when an interaction term with indigenous status is included. A statistically different association (p-value = 0.01) was observed for respiratory infections in indigenous people of 15.02% (95%CI: 3.73%, 27.54%) at a lag of 3 days while no association was evident for this condition in non-indigenous people at this lag (0.67%; 95%CI: -7.55%, 9.61%).

The point estimates for the effects in the other disease groups where not significantly different at the 95% confidence level; however the indigenous estimates were consistently higher than those for the non-indigenous population. The association of total respiratory admissions with same-day ambient PM_10 _in indigenous residents was much higher (9.40%; 95%CI: 1.04%, 18.46%) compared with the estimate for non-indigenous residents (3.14%; 95%CI: -2.99%, 9.66%). For asthma admissions and estimated ambient PM_10 _there was a non-significant estimated increase at a lag of 1 day: 16.27% (95%CI: -3.55%, 40.17%) for indigenous compared with 8.54% (95%CI: -5.60%, 24.80%) for non-indigenous people.

There were positive non-significant estimates for same day estimated ambient PM_10 _with COPD admissions in both groups. This is in contrast to negative associations with COPD admissions and lagged estimated ambient PM_10 _in both groups.

There were no clear associations with estimated ambient PM_10 _and total cardiovascular admissions or IHD. For non-indigenous admissions the estimated association with total cardiovascular admissions for ambient PM_10 _at lag 0 was -3.43% (95%CI: -9.00%, 2.49%) and the estimate for indigenous admissions was -3.78% (95%CI: -13.4%, 6.91%), although indigenous people did have positive (non-significant) estimates at lags 2 and 3.

## Discussion

We found generally positive associations between PM_10 _with total respiratory admissions, asthma and respiratory infections especially among indigenous people for total respiratory admissions (at lag 0) and respiratory infections (at lag 3). Negative associations were apparent between lagged PM_10 _and COPD. There were generally negative non-significant associations for cardiovascular outcomes in both population groups.

While we report our findings for several diagnostic sub-categories, the numbers in these groups, especially asthma and COPD, were much smaller and our confidence in effect estimates is greatest for the total respiratory and total cardiovascular classifications.

Our observed negative association with COPD admissions was unexpected as previous biomass studies have generally found strong positive associations with this outcome [33-36]. This could be due to hospital admission practices as in many instances patients presenting with exacerbations of asthma and COPD will be discharged home from the emergency department and therefore not be included in admissions data. However insufficient emergency department data were available for examination. Alternately it may be that persons with pre-existing chronic respiratory conditions take extra precautions with their care during days when PM levels are noticeably extreme.

In addition these counterintuitive results may be due to chance reflecting the low precision of our estimates due to the relatively small numbers of daily admissions. The lack of precision in our estimates could also be a function of other factors, such as uncertainty in exposure estimates, variation in population response, and even the lack of any association. However our findings are consistent with other studies of ambient biomass smoke and contribute to the limited evidence concerning the health effects of vegetation fire PM_10_.

A previous case-crossover study in Darwin had similar findings to this study with positive associations reported between observed ambient PM_10 _and respiratory admissions with Odds Ratio (OR) 1.08 (95%CI: 0.98, 1.18) and a tendency towards negative associations with cardiovascular admissions (OR 0.91; 95%CI: 0.81,1.02) [23]. Similarly, the estimates from that analysis for total respiratory admissions were also approximately double for indigenous rather than non-indigenous people.

A study in Christchurch, New Zealand, where ambient PM_10 _predominantly arises from the combustion of wood for domestic heating, found a 3.37% (95%CI: 2.34%, 4.40%) increase in total respiratory admissions per interquartile rise in ambient PM_10 _(IQR = 14.8 μg/m^3^) at a lag of 2 days [33]. That study found an association with admissions for heart failure but not other cardiac diagnoses, while a later study in Christchurch found no association with cardiovascular admissions [37].

In a study of the South East Asian forest fires of 1997 Mott *et al *[34] found large fire-period related increases in respiratory hospitalizations for asthma and COPD, ranging from 40–80% in adults but no association with cardiovascular admissions although people with pre-existing cardio-respiratory diagnoses were at greatest risk.

A recent study from Brisbane, Australia, directly compared the association between bushfire and non-bushfire derived particulates on total respiratory hospital admissions excluding influenza [3]. That study analyzed the PM_10 _distribution as a three-level factor with levels defined as low (< 15 μg/m^3^), medium (15–20 μg/m^3^) and high (> 20 μg/m^3^). They found that for an increase in same-day PM_10 _from low to high there was an increase in the relative risk for total respiratory hospital admissions of 19% (95%CI: 9%, 30%) whereas on non-bushfire days the associated increase was 13% (95%CI: 6%, 23%).

A similar study from Sydney, Australia, directly compared associations between cardio-respiratory hospitalizations and ambient PM_10 _derived from vegetation fire smoke with associations between these outcomes and ambient PM_10 _derived from other sources [4]. They apportioned ambient PM_10 _on vegetation fire days into particulate matter derived from burning biomass and particulates due to other sources. They found a 1.24% (95%CI: 0.22%, 2.27%) increase in relative risk for all respiratory admissions per 10 μg/m^3 ^increase in vegetation fire derived ambient PM_10 _at lag 0. Ambient PM_10 _due to other sources at lag 0 was associated with an increase in all respiratory admissions of 1.04% (95%CI: 0.02%, 2.07%) per 10 μg/m^3 ^increase. They also failed to find an association between cardiovascular outcomes and vegetation fire smoke in contrast to findings of a positive association between cardiovascular admissions and ambient PM_10 _from all other (non bushfire) sources.

The magnitude of the point estimates for all respiratory admissions from our study, the studies discussed above and several other studies of outpatient attendances for respiratory conditions in association with vegetation fires [33,34,38-41], are much greater than multi-city studies of associations between admissions for respiratory diseases (including asthma, COPD, and total respiratory admissions) with positive associations for a 10 μg/m^3 ^change in ambient PM_10 _of the order of just 1–1.5% [42,43]. Dominici *et al *2006 found similar associations of around 1% increase in respiratory admissions per 10 μg/m^3 ^change in PM_2.5 _[28]. The greater magnitude of adverse respiratory effects reported in studies specifically examining biomass smoke might reflect a true difference in the adverse outcomes associated with this source of PM. However, studies of biomass smoke are usually conducted in cities and towns with small populations, or around short episodes of extreme exposures, and their results inevitably are less precise than those from multi-city studies making direct comparisons difficult to interpret. Similarly, the absent or negative associations between biomass smoke and cardiovascular disease outcomes in our study and in three previous studies of vegetation fire smoke [4,23,34], might also reflect a different pattern of adverse health outcomes from biomass smoke. However these findings require replication as cardiovascular admissions have been clearly associated with ambient PM_10 _in many large studies, usually conducted in urban settings where fossil fuel combustion is a major source of PM [1].

The primary strengths of this study are the spatially homogenous population exposure to particulates across Darwin [17], the specific source from vegetation fires [15], the hospital data collection which represents the admissions patterns for the entire population of the city and the inclusion of details of indigenous status in the health records. These factors all minimized the problems of exposure and outcome misclassification inherent in population-level studies. Additionally, due to Darwin's tropical climate, there was minimal variation of daily temperature and humidity minimizing confounding by meteorological changes.

An important limitation of this study is the lack of air quality data, necessitating our use of an estimate based upon daily visibility. This inevitably will have introduced exposure misclassification bias limiting our ability to detect associations that might be present. In addition, because of the small population of Darwin, there were low numbers of daily admissions for cardio-respiratory diseases in spite of our relatively long 10-season period of data for analysis. This limited the statistical power and reduced the precision of our point estimates. However, population-level studies of the health effects of ambient biomass smoke have inherent limitations. Vegetation fire events affecting large populations are rare, unpredictable and often of short duration. In addition settings where biomass is the predominant source of ambient particulate matter tend to have smaller populations as larger cities will have a more complex mix of pollutants often dominated by fossil fuel combustion by industry and transport. For this reason the results from studies specifically examining vegetation fire smoke pollution will almost inevitably have greater technical challenges than studies examining ambient PM regardless of source.

A key reason why PM_10 _effect estimates may differ by region is the different sources and resulting chemical composition of particles, such as the biomass burning noted here. Most of the literature concerning chemical composition compares different size classes, such as PM_10_, PM_2.5_, PM_2.5–10 _and PM_1 _and while all size classes have been associated with adverse heath outcomes, smaller particles have generally been found to be relatively more toxic [1]. Our study could not examine different size classes however, a detailed study of PM in Darwin during 2004–5 found that the total PM_2.5 _comprised on average 56% of the total PM [44].

In addition to the different ratios of size fractions, bushfire derived PM is associated with a distinctive suite of toxic co-pollutants including metals, organic and inorganic compounds [9]. In vitro studies have demonstrated that different chemical compositions induce different cellular responses [45]. Moreover a few epidemiological studies have apportioned total PM according to a range of sources (such as biomass, crustal and motor vehicles) have found that the magnitude of a range of different clinical outcomes were associated with different exposure sources. A study of hospitalizations in Copenhagen, Denmark found respiratory outcomes to be predominantly associated with biomass particulates and crustal and secondary particulate sources with cardiovascular outcomes [7]. However in that study asthma was more closely associated with markers of car exhaust. A study of mortality in Phoenix, USA found that secondary sulfate, traffic, and copper smelter-derived particles were most consistently associated with cardiovascular mortality while biomass derived particulates were not [8]. These source apportionment studies are compatible with the few studies, including ours, that have specifically examined biomass smoke derived PM.

Our study also compared rates of admissions between indigenous and non-indigenous subpopulations finding the suggestion of disproportionate burdens of health effects due to the seasonal fire smoke pollution; especially a statistically different association between PM_10 _and admissions for respiratory infections three days later. This is consistent with the only previous study examining this issue in Australia [23]. Many factors could contribute to this including excess socio-economic disadvantage, chronic cardio-respiratory diseases and diabetes [21] which all modify the effects of ambient PM_10 _on cardio-respiratory admissions [20]. Other factors could include reduced access to health services and therefore early management of chronic conditions [46], and different patterns of smoking, physical activity or diet among this population sub-group [21]. In addition, the two populations have differing age structures, with a greater proportion of people over sixty-five in the non-indigenous group, and a greater proportion of children less than 15 years in the indigenous group [19]. The former factor could result in an underestimate of the difference between the two population groups, while the latter could have contributed to the differences in respiratory infections observed between the two groups. We have attempted to control for this age-structure effect by including a term for indigenous status which should capture this. Residential segregation is less likely to explain the difference in this setting as exposure is relatively uniform across the city [17].

## Conclusion

Our results suggest associations between vegetation fire smoke and daily hospital admissions for respiratory diseases that were stronger in indigenous people. The analysis found approximately three-fold higher associations between same-day estimated ambient PM_10 _and total respiratory admissions in indigenous people than non-indigenous people. This has implications for local public health policy and practice, such as the identification of sensitive sub-groups, the setting of air quality guidelines, targeting of public health messages in relation to air pollution and the regulation of deliberate burning practices [22].

This is an important research area to pursue. With global change bringing changes in vegetation burning regimes and increasing population exposures to pollution from vegetation fires, understanding and managing the health impacts of biomass combustion smoke will become an increasingly important public health activity.

## Abbreviations

CI: Confidence interval; COPD: Chronic Obstructive Pulmonary Disease; ICD10: International Classification of Diseases, 10th Revision; ICD9: International Classification of Diseases: 9th Revision. IHD: Ischemic Heart Disease; OR: Odds ratio; PM: Particulate Matter; The aerodynamic diameter of the particles is shown by the additional of the size range in microns as subscripts. For instance PM with diameter less than or equal to 10 microns is PM_10, _PM with diameter less than or equal to 2.5 microns is PM_2.5 _and so forth.

## Competing interests

The authors declare that they have no competing interests.

## Authors' contributions

ICH carried out the analysis and drafted the manuscript. FHJ conceived the study and helped to draft the manuscript. GGM provided theoretical and conceptual guidance and helped to draft the manuscript. All authors have read and approved this version of the manuscript.
